# Parenthood and mental health throughout the life course: A total population study

**DOI:** 10.1007/s00127-026-03091-7

**Published:** 2026-04-29

**Authors:** Maria Lyster Andersen, Hans Fredrik Sunde, Rannveig Kaldager Hart, Fartein Ask Torvik

**Affiliations:** 1 Centre for Fertility and Health, Norwegian Institute of Public Health, Myrens Verksted 3L, Oslo, 0476 Norway; 2 Promenta Research Center, Department of Psychology, University of Oslo, Forskningsveien 3A, Oslo, 0373 Norway; 3 Department of Health Management and Health Economics, University of Oslo, Forskningsveien 3A, Oslo, 0373 Norway

**Keywords:** Mental health, Parenthood, Social inequalities, Childlessness

## Abstract

**Purpose:**

Do parents or childless adults have better mental health? The answer could vary by life phases, but past studies exploring the link between parenthood and mental health have not considered the entire life course. This study investigates the association between parenthood and mental health in an entire population and further looks at how parenthood interacts with factors such as age, gender, and socioeconomic status.

**Methods:**

We used comprehensive Norwegian register data covering the entire population aged 31–80 years old (*N* = 2,234,087) to explore the relationship between parenthood and mental health conditions from 2006 to 2019. We used logistic regression models for population-wide analyses and conditional logistic models for analyses of discordant same-sex sibling sets and monozygotic twin pairs.

**Results:**

Parenthood was associated with a lower risk of mental disorders (OR 0.67, 95% CI 0.67–0.68 for women; OR 0.60, 95% CI 0.59–0.60 for men), including depressive and anxiety disorders. Fathers also had a minor reduction in risk of symptoms related to mental illness (OR 0.98, 95% CI 0.97–0.98), while mothers had a slightly elevated risk of symptoms (OR 1.09, 95% CI 1.09–1.10). Overall, mental health disparities between parents and non-parents persisted throughout life and were more pronounced among men and those with low educational attainment. Our findings were consistent in sibling- and twin-matched analyses.

**Conclusions:**

Our findings highlight parenthood as a significant indicator of mental health inequalities throughout adulthood and old age.

**Supplementary Information:**

The online version contains supplementary material available at https://doi.org/10.1007/s00127-026-03091-7.

## Introduction

Mental disorders constitute a major public health challenge, accounting for the most working years lost in the Norwegian population [1] and ranking among the leading causes of disease burden worldwide [2]. However, the risk and distribution of mental disorders are not equally distributed across populations [3, 4]. Consequently, understanding how mental disorders relate to sociodemographic factors across the life course is important. Whereas associations with psychosocial factors such as sex and educational attainment have been extensively documented [3, 5–7], links with parenthood have so far been underexplored.

This gap in the literature is especially noteworthy given the parallel trends of rising mental health challenges and declining fertility rates observed in many high-income countries. There have been considerable changes in fertility behaviour over the last decade, as people are having fewer children and postponing their first birth [8]. As childlessness has become increasingly prevalent [9], parenthood — and the absence thereof — could serve as an important sociodemographic marker for understanding mental health disparities. Despite this, the implications of parenthood for mental health across the *entire* life course have received limited attention.

Fundamentally, differences in mental health between parents and childless adults can be the result of two main processes. First, mental health status can affect both whether and when an individual becomes a parent. Studies on health-based selection into parenthood find that the presence of mental disorders early in life is predictive of later fertility. For instance, depression has been linked to lower fertility and earlier timing of parenthood in both Finland [10] and Norway [11]. A large population study from Finland and Sweden also found that across all disease categories, mental-behavioural diseases were the most strongly associated with later-life childlessness [12]. Many of the traditionally used markers of socioeconomic status such as education or income are also related to both selection into parenthood [13–15] as well as mental health outcomes [16]. This underscores the importance of considering not only health-based selection but also how socioeconomic characteristics mediate both parenthood and mental health outcomes.

Second, being a parent can affect mental health, and this effect can vary with the child’s (and thus the parent’s) age, as well as with the sex of the parent. It is well documented that there is increased risk of mental disorders such as depression and anxiety in the ante- and post-natal phase [17–19]. However, the post-natal phase is a formative but short phase of parenting, and our knowledge of how the relationship between mental health and parenting varies in a wider age range is much more limited. In the longer run, parenthood could have positive and negative impacts on mental health. While parenting can be inherently stressful, most parents find that parenthood gives meaning and provide joy [20], potentially buffering against mental health challenges.

Past research on the association between parenthood and mental health has found diverging results [21–27]. A cross-sectional study from Germany found that parenthood was associated with better mental health, in particular among men, when comparing those living with at least one child to childless individuals [24]. Yet, a similar cross-sectional study from Norway found that when comparing empty-nest parents to childless adults, there were only small positive associations with women’s well-being, and no association with men’s well-being [22]. Another Norwegian study looking at parenthood and symptoms of anxiety or depression in a large population-based cohort found no differences between parents and non-parents [26]. This study also focused on a narrow age-range (30–49), excluding most empty-nesters. Finally, a cross-sectional U.S.-based study found a negative link between parenthood and mental health, with some types of parenthood being associated with especially high levels of depression (e.g. parents of minors) [21].

Fewer studies have examined the mental health impact of parenthood while accounting for the previously discussed potential selection effects. A small (*n* = 7095) UK-based longitudinal cohort study found modest mental health benefits of parenthood [28]. However, they also noted that there was significant heterogeneity in the results based on factors such as timing of first birth, with older parents having better mental health. Importantly, the last follow-up in this study was at age 32, only allowing for the detection of short-term health effects. A similarly designed Swedish study (*n* = 1001) following individuals up to age 43 found a positive mental health effect of children for women but null-effects for men [29]. Interestingly, larger register-based studies from comparable high-income countries have found opposite effects. When studying German claims and survey data, motherhood was found to increase the likelihood of mental illness and antidepressant use at least four years into the post-birth period [30]. A similar pattern was found for other stress-related outcomes such as headaches or painkiller use. Finally, an event-study using Austrian and Danish administrative data found a mental health penalty of parenthood for both women and men [31]. As the negative effects were significantly larger for women, the transition to parenthood created a wider gender gap in the mental health of parents. Notably, the mental health penalty was also especially large for parents who were young at the transition to parenthood or had low education. While the Austrian analysis was limited to a 9-year follow-up length, the mental health effects were persistent for the entire 25-year follow-up window in the Danish data.

In sum, past studies have found diverging results when investigating the link between parenthood and mental health. The transition to parenthood might negatively affect mental health, yet cross-sectional studies find both negative and positive associations between parenthood and mental health. Importantly, these diverging findings likely reflect how the strains of parenthood are unequally distributed across social groups and ages. However, we are not aware of any studies that look at an entire population of parents and childless adults, covering the entire life-course. Diverging results could also stem from variation in how previous studies operationalise mental health. Previous work variously investigates diagnoses of mental disorders, symptoms of anxiety and depression, or even subjective wellbeing. Although related, such measures are qualitatively different [32], and their associations with parenthood could vary. The diverging findings may also reflect a fundamental challenge: disentangling the effects of parenthood from the genetic and environmental factors that influence both selection into parenthood and baseline mental health risk. Factors such as one’s family and upbringing, genetic predispositions for psychopathology, and cognitive abilities can confound standard observational estimates [33–36]. Although previous studies have adjusted for many measured confounders, residual confounding from unobserved factors remains. We address this by employing a sibling and discordant monozygotic twin design. By comparing twins who share genetics and upbringing but differ in mental health, we can adjust for some of the confounding factors that bias conventional estimates and more credibly estimate the association between parenthood and mental health [37].

Consequently, this study aims to document group differences in mental health by parenthood across the life course, controlling for both observed and unobserved confounders. To this end, we used population-wide health care data covering the entire Norwegian population. We addressed three research questions:

**1) How is parenthood associated with mental health among men and women across the life-course?**

**2) Do differences in mental health between parents and non-parents vary by socioeconomic background?**

**3) How is parenthood associated with different kinds of diagnoses and symptoms recorded in primary care?**

## Methods

### Sample

Our study population is based on the Norwegian National Population Register. This register includes anyone who has been registered as residing in Norway since the 1960 census and contains information about time of birth, death, and parent identifiers until the end of 2020. This was linked to educational and socioeconomic register data and diagnostic data from medical records covering the years 2006–2019. The data from these different sources were joined using pseudonymised individual-specific personal identification numbers. To minimise incomplete data, we limited our analyses to only include Norwegian-born individuals who also were registered as residing in Norway during the observation period (2006–2019). To ensure that individuals belong to only one group (i.e., parent or childless), we focus on completed fertility at age 45. Consequently, only those born in 1975 or earlier are included. This leaves in total 28,823,322 person-years stemming from 2,234,087 unique individuals in our dataset.

Certain genes or environmental factors such as upbringing or the relationship with one’s own parents might influence both the risk of mental disorders and the likelihood of having children. To adjust for these potential genetic and environmental effects shared between siblings that confound the relationship between parenthood and mental health, we also used parent identifiers to create a sub-sample of same-sex full-siblings in the population. When limiting the sample to only those with at least one full same-sex sibling, we have 13,036,358 observations based on 434,070 unique sets of same-sex siblings (202,613 female sets and 231,457 male sets). To achieve full adjustment for genetic factors, we also identified 2,132 female and 2,380 male monozygotic twin pairs from the Norwegian Twin Registry.

## Measures

### Parenthood and age at first birth

Data from the Population Register is used to identify all first births in the population. We defined parents as individuals who were registered with a first child before or at age 45, while we defined those registered without any children by age 45 as childless.^1^ Notably, the registers only identify legal parents, and most often, this means biological parents. However, adopted children and children born with the help of assisted reproductive technology are also included. We also use the Population Register to identify the individual’s age at first birth. Women were classified as having an “early” first birth if it occurred before age 25 and as “advanced” if at age 33 or older. For men, the respective cut-offs were 27 and 35. We also record the number of children at age 45. These cut-offs were chosen as they are easily interpretable whilst also capturing the overall distribution of age at first birth for many cohorts [39]. As age at first birth varies by cohort, we have tested alternative specification using indicators for being in the top/bottom quartile of the age distribution of your birth cohort and gender. This does not substantially change the results.

## Mental disorders and symptoms

Information about primary health care utilisation comes from the Norwegian Control and Payment of Health Reimbursements Database (KUHR), which was established in 2006. The KUHR database contains information from different contact points within the health care system, with most of the data coming from general practitioners. When practitioners submit their reimbursement claims to the Norwegian Health Economics Administration (Helfo), they use the International Classification of Primary Care (ICPC-2) system to register the reason for a patient’s visit [40]. Codes P01-P29 are used to indicate psychological symptoms, while codes P70-P99 refer to diagnoses of mental disorders.^2^ As general practitioners are required to submit these forms to receive reimbursements, it is unlikely that visits are under-reported in our data. However, the oldest age groups in our dataset are likely underrepresented in reported visits, as services provided in institutional settings such as nursing homes are not captured in the KUHR system.

We rely on primary care data rather than specialist care data because it allows us to examine the association between parenthood and mental health in the full population. Only a small share of the population receives specialist care for mental health problems [41], so analyses based solely on specialist records would capture a selected subset of the affected population. Moreover, because primary care is the first point of contact, individuals who receive specialist care are nearly always also recorded in primary care data [42, 43]. Consistent with this, prior studies from Norway document that mental disorder diagnoses recorded in primary and specialist care capture largely the same phenomena, albeit at different thresholds of severity [44].

Mental health outcomes are measured through registered visits to health care services in the KUHR database using the aforementioned codes. We created two binary variables, each reflecting whether an individual had at least one visit to primary care services related to a psychological symptom or a diagnosed mental disorder in a given year of our observation period. A value of 1 indicates at least one visit in that year, while a value of 0 indicates no visits. Additional binary variables are created for specific common disorders and symptoms such as depression, anxiety, and sleep problems.

Ideally, we would also want to include a measure of previous mental health. However, as the primary care register only is available from 2006 onwards this is not possible.

## Educational attainment

Data on educational attainment was obtained from Norway’s National Education Database (NUDB), operated by Statistics Norway. The Norwegian Standard Classification of Education (NUS2000) is used to group individuals based on their highest level of completed education by each observation year. We used four categories: lower secondary schooling or less (i.e. compulsory education), upper secondary schooling, bachelor’s degree, and master’s degree or higher.

### Partnership status

Information on partnership status comes from the Population Register. The partnership status registered the year an individual turns 45 was used to classify people as either married or non-married at age 45. We only considered marriage as this is the most common formalised form of partnership, and the recorded data on non-marital partnership is less reliable for couples without shared children. We measure marital status at age 45 because this matches our measure of parenthood and because these data are available for all individuals in the sample. In a robustness test, we use a time-varying measure of marital status including all types of registered relationship statuses: unmarried, married, widow/widower, divorced, separated, registered partner, separated partner, divorced partner, and surviving partner. Results using this measure, shown in Supplemental Figure A3, are substantially similar.

### Statistical Analysis

We first calculated age-specific prevalence rates for diagnosed mental disorders and psychological symptoms separately for men and women, stratified by parenthood and partner status at age 45. Rates were also calculated based on age at first birth and number of children at age 45. To estimate the overall association between parenthood and mental health across all ages, we used logistic regression models with psychological symptoms or mental disorders as the primary outcome variables. In our full model, we also include covariates that could confound the relationship between parenthood and mental health. These include age (in the observation year), educational attainment (highest achieved by the observation year), partnership status at age 45, and the year of observation. As both parenthood and prevalence of mental disorders vary between the genders, we analysed men and women separately. Consequently, mental health outcomes are modelled as a function of parenthood though the following two logistic regression equations:

1 $$\begin{gathered} In\left( {\frac{{P(Y = 1)}}{{1 - P(Y = 1)}}} \right) = \alpha + Parent\beta _{1} \hfill \\ \,\,\,\,\,\,\,\,\,\,\,\,\,\,\,\,\,\,\,\,\,\,\,\,\,\,\, + Age\beta _{2} + Z_{k} \beta _{3} + \varepsilon \hfill \\ \end{gathered}$$

In equation 1, *Y* is either a mental disorder or symptoms, and *P(Y=1)* is the probability that a mental disorder or symptoms are present. The full expression on the left-hand side is consequently the log-odds of the probability of *Y* being equal to 1. *α *is the estimated intercept, *Parent* is a binary variable equal to 1 if one has children at 45, and *Z*_*k*_is a vector of the remaining control variables (i.e., marital status, educational attainment, year, age at first birth). Our main coefficient of interest is *β*_1_. This logistic regression model is estimated for our population analysis. In order to further control for genetic and shared environmental factors between siblings, a similar model is estimated through conditional logistic regressions for our sibling-matched and monozygotic twin sub-samples.

2 $$\begin{gathered} In\left( {\frac{{P(Y_{{ij}} = 1)}}{{1 - P(Y_{{ij}} = 1)}}} \right) = Parent_{{ij}} \beta _{1} \hfill \\ \,\,\,\,\,\,\,\,\,\,\,\,\,\,\,\,\,\,\,\,\,\,\,\,\,\,\,\,\,\,\,\,\,\,\,\, + Z_{{k_{{ij}} }} \beta _{2} + \gamma _{j} + \varepsilon \hfill \\ \end{gathered}$$

In equation 2, *P(Y*_*ij*_*=1)* is the probability that the dependent variable *Y* is equal to 1 for individual *i* in sibship *j*. The full expression in the left-hand side is the log-odds of the probability of Y_*ij*_equalling 1. Parent_*ij*_is again a binary variable for individual *i* in sibship *j*, that equals 1 if one is a parent. γ_*j*_represents the stratum-specific effects, i.e., the individual intercepts for each sibling or twin group. This model is applied only to sibling or twin groups that are discordant on the outcome, meaning that at least one individual has the disorder/symptom and at least one does not.

All statistical analyses were done using Stata MP17 with the logistic and clogit commands.

## Results

The proportion of childless individuals at age 45 was higher among men (16.2%) than women (9.9%). Descriptive statistics for the entire population as well as a yearly sub-sample of 45-year-olds are presented in Table A1 (see supplements).

**Fig. 1:**
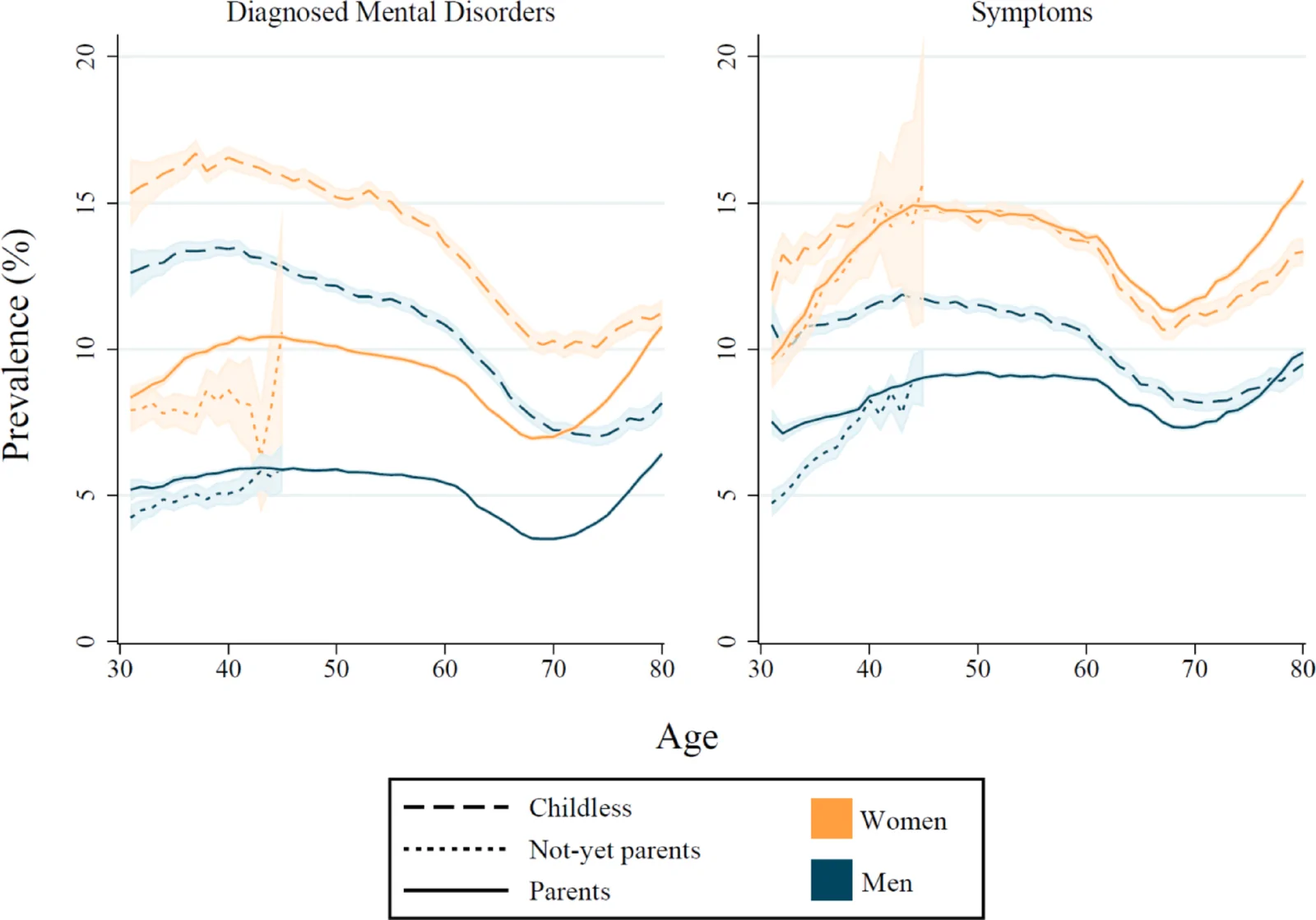
Prevalence of mental disorders and mental symptoms by age, gender, and parenthood-status. Shaded areas represent 95% confidence intervals

### Mental health and parenthood across the adult life course

Figure 1 shows the unadjusted relationship between parenthood and mental health over the adult life course for men and women, with diagnosed mental disorders in the left panel and symptoms in the right panel. Up to age 45, we distinguish between not-yet-parents (those who have children later but are currently not parents at the observed age) and consistently childless. Starting with diagnosed mental disorders (left panel), we find that across all ages, mothers had a lower risk of mental disorders than childless women, and fathers had a lower risk of mental disorders than childless men. For both sexes, the difference appeared greatest earlier in adulthood and then reduced at older ages. Men and women who were not yet parents had even lower levels of mental disorders than their counterparts who already were parents. Consequently, the largest difference in mental disorder risk observed was between not-yet-parents and childless individuals.

As for symptoms (right panel), the same pattern holds for men: across almost all ages, fathers had lower levels of symptoms than childless men, albeit with a smaller difference than found for disorders. The lowest levels of symptoms were found among young men who had not yet entered parenthood. For women, a different picture emerges: in early adulthood, the consistently childless women had the highest level of symptoms. Meanwhile, mothers and not-yet mothers follow each other closely in the same period. From age 45 to around 60, the level of symptoms was similar among mothers and childless women. Finally, after age 60, mothers had more symptoms than childless women.

**Fig. 2:**
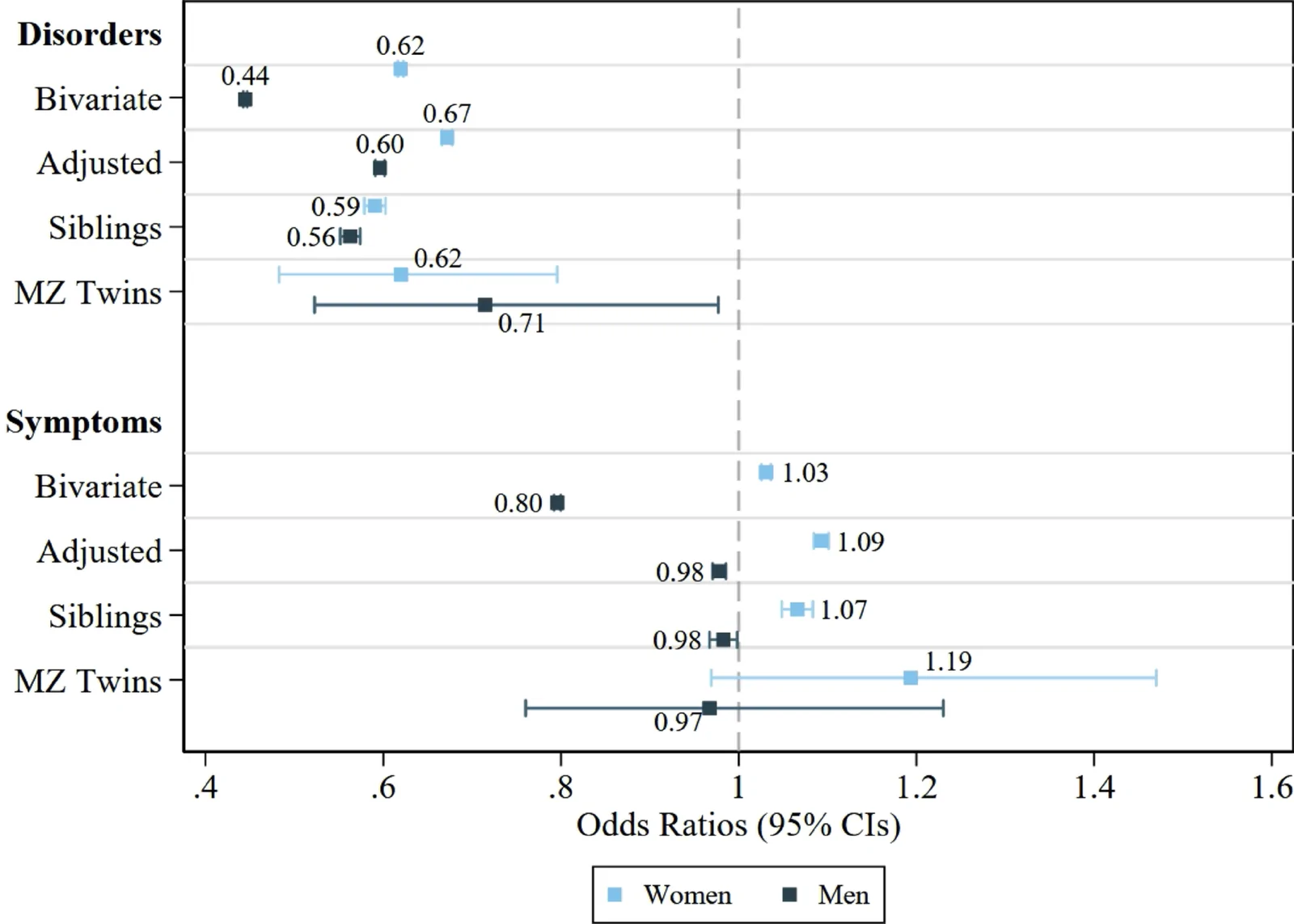
Summary of the different association models estimated between parenthood (parent = 1) and mental health

Figure 2 presents the estimated associations between parenthood and mental disorders, as well as parenthood and symptoms/complaints related to mental health. Detailed results are presented in the supplementary materials (Table A2 and Table A3). We found a significant relationship between mental disorders and parenthood in all of the eight specified models. In both the bivariate and adjusted population models, we found that parents had lower odds of mental disorders. In the fully adjusted model, mothers had 33% lower odds of disorders, whereas fathers had 40% lower odds of disorders. In the sibling-matched analysis we found that mothers had on average 41% lower odds of mental disorders (95% CI 0.58–0.60), while fathers had 44% lower odds (95% CI 0.55–0.57). When limiting our sample to only monozygotic twin pairs who were discordant in terms of mental disorders, we still found significantly lower odds of mental disorders for both fathers (OR 0.71) and mothers (OR 0.62).

We also found a significant relationship between mental symptoms/complaints and parenthood in all but two of the eight specified models. However, these went in the opposite direction for men and women. In the fully adjusted population model, we found that mothers had 9% higher odds of symptoms, while fathers had 2% lower odds of symptoms compared to the childless. In the sibling-matched analyses we found that mothers had 7% higher odds of symptoms (95% CI 1.05–1.08), while fathers had 2% lower odds (95% CI 0.97–0.99). When limiting our sample to only discordant monozygotic twin pairs, we found no significant difference in the odds of symptoms. Nonetheless, the direction of the associations remained consistent with the other models, though with reduced precision in the estimates.

When estimating the same fully adjusted models as in Fig. 2 stratified by age, we found similar results (see Fig. A1 in supplements). After adjusting for factors such as education or partnership, parents had significantly lower risk of mental disorders across all ages. For both men and women, the largest difference in terms of mental disorders was seen early in adulthood. Furthermore, we found that especially mothers were prone to psychological symptoms, with an increased risk of symptoms present after age 40. Unlike in the unstratified analyses, some fathers also had an elevated likelihood of psychological symptoms, which appeared at later ages (ages 55 and above).

### Variations in associations by socioeconomic status

**Fig. 3:**
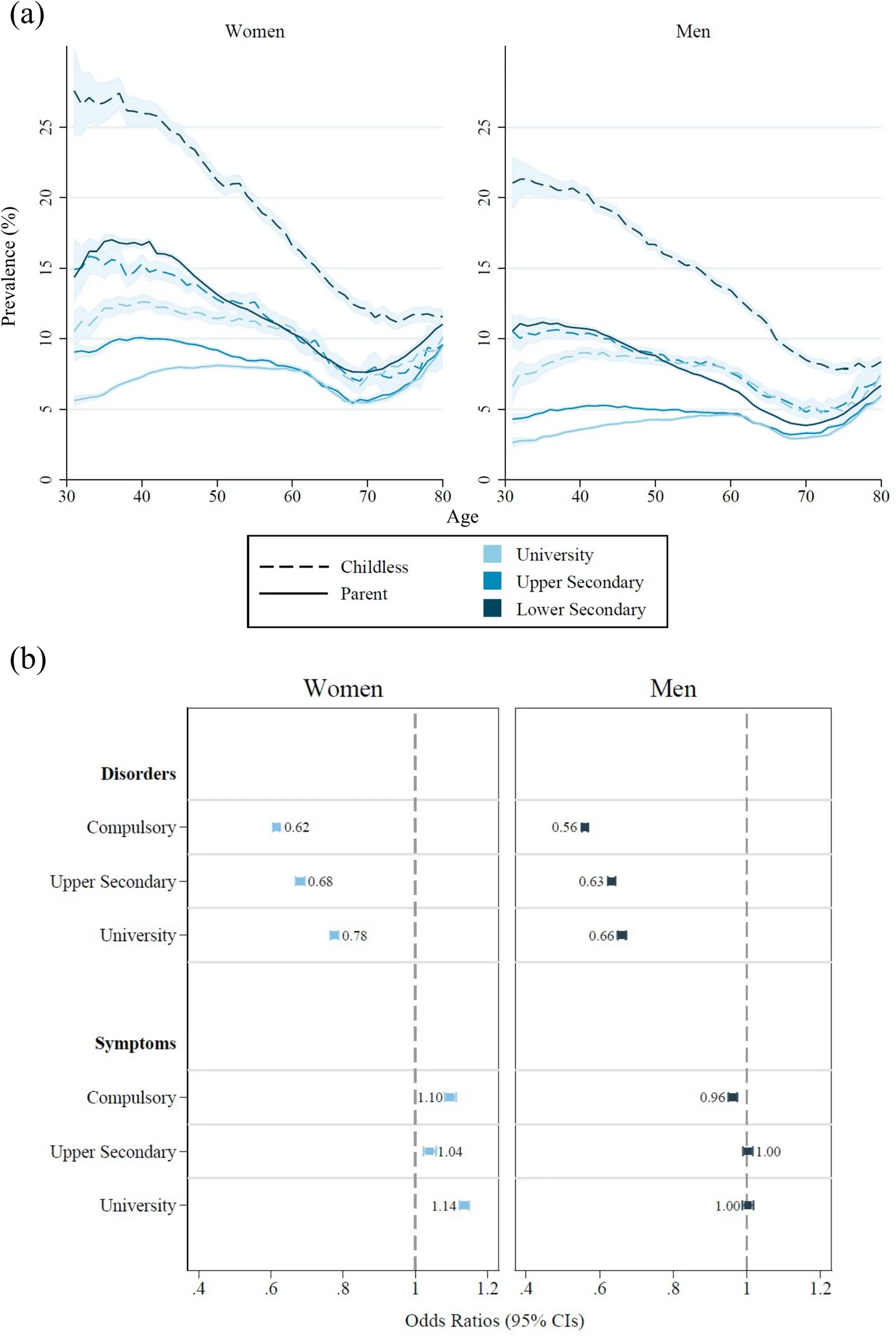
Panel **(a) **shows the prevalence of mental disorders (in percent) across age by parenthood and educational attainment. Shaded areas represent 95% confidence intervals. Panel **(b)** shows the estimated odds of mental disorders or symptoms depending on parenthood (parent=1) by age 45 for different educational levels. Fully adjusted models, controlling for age, year, age at first birth, and marital status

As shown in Fig. 3 panel (a), the prevalence of mental disorders was by far the highest among childless men and women with only compulsory (i.e. lower secondary) education. This pattern persisted across the entire life course. However, within both the group of childless adults and parents, we see a clear educational gradient. The lowest prevalence of mental disorders is seen among those with university education, and the highest among those with compulsory education. At the same time, while parents generally have lower levels of mental disorders, parents with the lowest level of educational attainment are more similar to the childless. While we see the largest differences in mental health between early adulthood and middle age, even at ages 70 and above there are notable differences in the mental health of the various groups.

Next, we studied associations between parenthood at age 45 and mental health in different educational groups. The results are shown in Fig. 3 panel (b). Parenthood was associated with a lower risk of mental disorders in all educational groups, but the difference between parents and non-parents was largest among those with only compulsory education (OR 0.62 for women and 0.56 for men). For symptoms, the associations were smaller and did not uniquely run in one direction.

We found similar results when stratifying by the other markers linked to socioeconomic status (marriage, number of children, and age at childbirth). These results are presented in Figure A2 (supplements). Specifically, parenthood was significantly associated with risk of mental disorder among both married and unmarried individuals; however, the association was substantially stronger among unmarried individuals.

### Specific diagnoses and symptoms

**Fig. 4:**
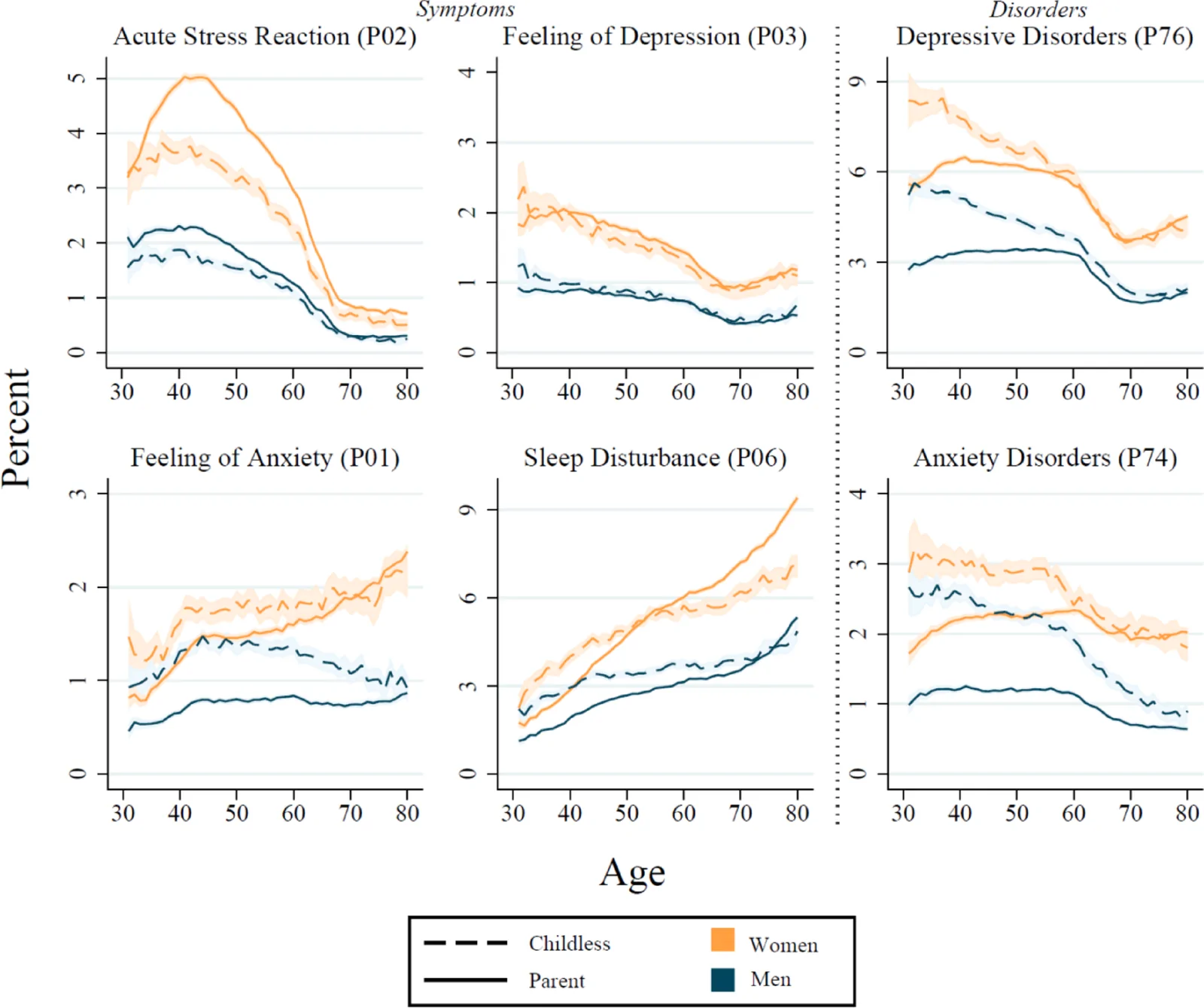
Prevalence of various mental disorders and symptoms by age, gender, and parenthood (at age 45). Shaded areas represent 95% confidence intervals

Figure 4 shows the prevalence of specific diseases and symptoms among parents and non-parents across the life course. We have focused on diseases or symptoms that are common (> 1%) in most age groups. The ICPC-2 codes are differently associated with parenthood. For example, while the childless had on average a higher prevalence of depressive and anxiety disorders, there was a less marked difference when looking at feelings of depression. Conversely, the childless still had a higher prevalence of feelings of anxiety, especially childless men. On the other hand, parents, and especially mothers, had a much higher prevalence of acute stress reactions. This difference was especially large around ages 40–50.

**Fig. 5:**
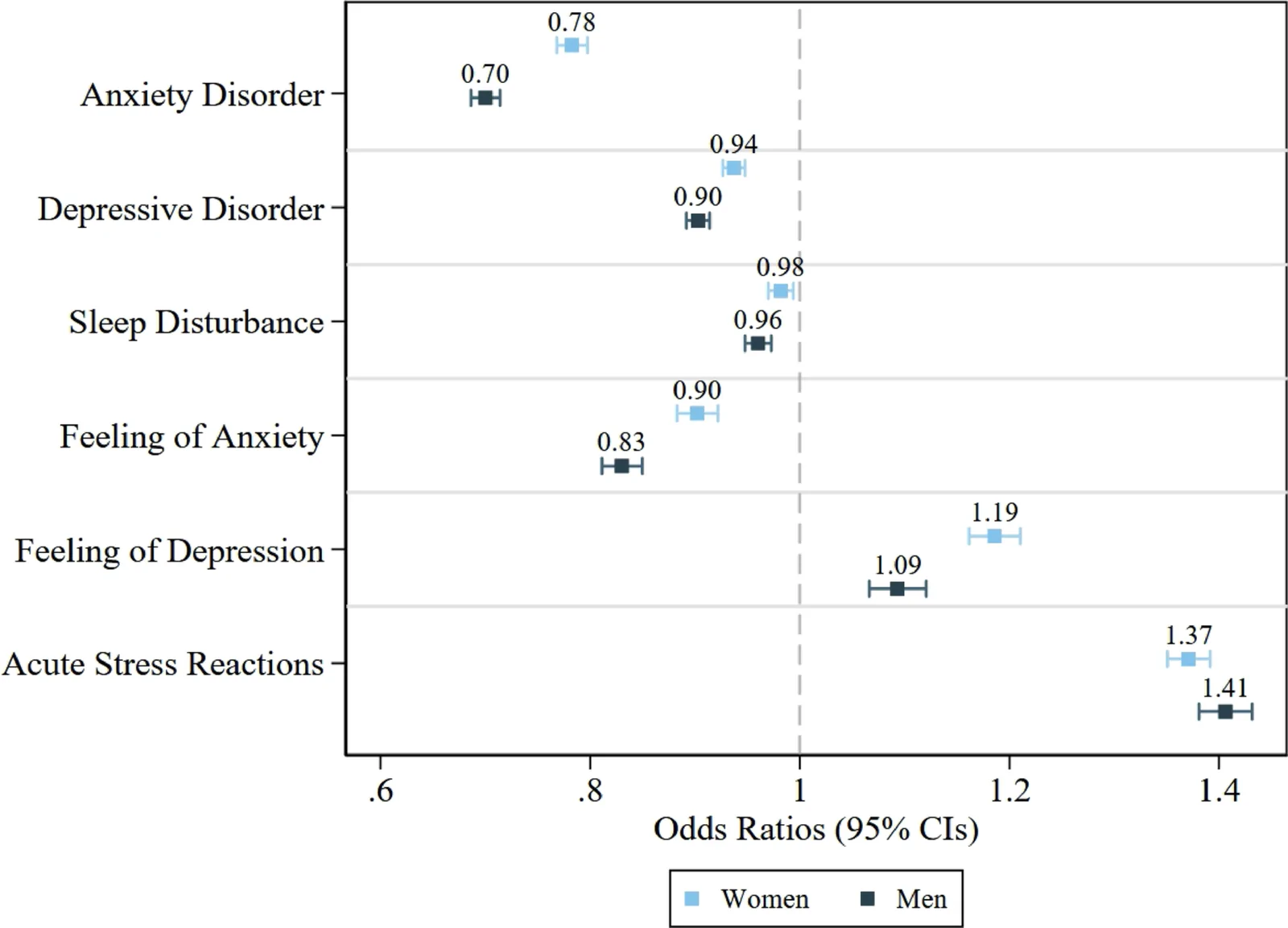
Associations between specific mental disorders/symptoms and parenthood (parent = 1). Fully adjusted models, controlling for age, year, age at first birth, education, and marital status. ICPC-2 codes used are P01 (feeling of anxiety), P02 (acute stress reaction), P03 (feeling of depression), P06 (sleep disturbance), P74 (anxiety disorder), and P76 (depressive disorder)

We then estimated the association between each symptom or diagnosis and parenthood separately, with the results presented in Fig. 5. In these fully adjusted models, we also found that parenthood was not consistently associated with adverse mental health outcomes. While parents had lower odds of both anxiety (OR 0.78 for women and 0.70 for men) and depressive disorders (OR 0.94 and 0.90), certain symptoms were positively associated with parenthood. Both mothers and fathers had higher odds of feelings of depression (OR 1.19 and 1.09) and acute stress reactions (OR 1.37 and 1.41) when compared to otherwise similar childless adults. For the two other most prevalent symptoms, feelings of anxiety (OR 0.90 and 0.83) and sleep disturbance (OR 0.98 and 0.96), parenthood was again associated with lower odds.

## Discussion

We examined the association between parenthood and mental health among Norwegian-born individuals aged 31–80 from 2006 to 2019, providing a comprehensive life-course perspective that reconciles prior mixed findings. Our results revealed that overall, parents had a lower likelihood of mental disorders compared to childless individuals. With regards to symptoms, mothers had a slightly higher risk, while fathers had a marginal reduction in risk. The parenthood advantage in mental disorders persisted throughout adulthood but diminished in the very oldest age groups. We also found a gradient in the link between mental health and parenthood across socio-economic status, defined by educational attainment. With regard to specific conditions, parenthood was associated with a lower risk of depressive disorder, anxiety disorder, sleep disturbance, and feeling of anxiety. Simultaneously, parenthood was associated with a higher risk of depressive feelings and stress reactions.

Past research on parenthood and mental health has provided mixed results, with many cross-sectional studies finding positive or no associations [22, 24, 26] and larger longitudinal studies finding a negative relationship between parenthood and mental health [23, 30, 31]. Our findings help explain how many of these different findings may be true at the same time. In our study, both mothers and fathers had significantly lower risk of mental disorders compared to childless adults. At the same time, we found that the group of not-yet parents had an even lower risk of mental disorders. This suggests that becoming a parent may increase health care use related to mental disorders – in line with the findings from previous longitudinal studies. However, even with this increase, overall, there remained a large and significant difference in mental disorder risk between parents and childless individuals. Consequently, while parenthood may worsen parents’ mental health and/or increase their health care use, parents still have *significantly* lower risk of mental disorders throughout life. Furthermore, as we found that parenthood was associated with a lower risk of all disorders but a higher risk of certain symptoms (especially for women), cross sectional studies might find different associations depending on what specific mental health outcome is studied and within which context.

In the Nordic context, both fathers and mothers balance paid and unpaid work, leading to a potential “time squeeze” [45, 46]. This time squeeze is especially prominent early in parenthood when childcare is the most time-intensive [47]. Consequently, the stressors of parenthood might change as the children age [48], altering the association between mental health and parenthood. However, there are no quantitative studies examining how the relationship between parenthood and mental health varies across the life course. Our study addresses this gap, revealing a consistent mental health advantage for parents throughout most of life, even during the most demanding years. We find similar results also when adjusting for confounders such as education and partnership, and within monozygotic twin pairs. The slightly attenuated estimates in the twin analyses indicate that part of the fertility–mental health association reflects confounding from genetic and shared environmental factors. However, taken together with our other models, our findings suggest that socioeconomic factors alone cannot fully explain the association between parenthood and mental health. Interestingly, we found the largest difference in mental disorder risk early in adulthood and then observed some convergence in risk between parents and childless adults in the oldest age groups. Thus, while the early years of parenthood might be the most stressful, this stress does not elevate mental disorder risk to the level seen in childless adults of the same age. At older ages, we believe the convergence in risk reflects mechanisms such as survivorship bias [49], as childless individuals who live longer might be overall healthier [50]. The presence of children and additional extended family-members could also result in increased help-seeking among elderly parents compared to childless individuals [51]. Later in life, parents may also experience illness or death among their children, which can negatively impact their mental health [52, 53]. Lastly, as children age and move out, parents might increasingly rely on broader social networks for emotional and practical support, reducing the mental health differences between parents and childless individuals.

While parenthood overall was associated with better mental health, we observed significant disparities in the link between mental disorders and parenthood across socioeconomic groups. Although educational attainment attenuated the relationship between parenthood and disorders, the association persisted across all educational levels and appeared strongest among those with the lowest educational attainment. This finding suggests that childlessness compounded by low educational attainment may result in an accumulative disadvantage with respect to mental disorder risk. One potential explanation for these disparities lies in differences in rates of involuntary childlessness across educational groups. Historically, women with higher education have been more likely to opt out of parenthood, perhaps prioritising career aspirations [54]. However, this dynamic appears to be shifting among younger cohorts, where increasing numbers are pursuing parenthood alongside full-time careers [55]. Furthermore, as the connection between education and voluntary childlessness does not exist for men [56], the strong link between parenthood and mental health for those with lower educational attainment is likely driven by additional factors. For example, individuals with low education and more uncertain labour market attachment [57] may derive a greater sense of meaning from the social networks and social capital associated with parenthood [58, 59]. The strong link between parenthood and mental health among those with low education might also result from the well-established link between educational attainment and overall health [60–62]. Those with low education are at higher risk of having overall poor health, which might include both infertility as well as mental health issues. Poor health might also make it more difficult to find a partner [63], thus increasing the likelihood of childlessness. Similarly, marital status attenuated the association between parenthood and mental health, consistent with well-established links between partnership status and health outcomes [64–67]. Nevertheless, stratified analyses revealed a significant association even within the married sample, suggesting that the mental health implications of parenthood extend beyond selection into partnership.

As pointed out, parenthood was not linked to a decreased likelihood of all negative mental health outcomes. Mothers faced a slightly higher risk of mental illness symptoms, while fathers showed a minimal decrease in risk. This gender difference is possibly due to the unequal distribution of child-rearing responsibilities [68]. Parenthood may also interact with gender to shape health-seeking behaviour. However, prior research on the mental health penalties of parenthood suggests that gendered differences in help-seeking do not fully account for the observed gender gap [31]. Furthermore, specific symptoms such as feelings of depression and acute stress reactions were overall more prevalent among parents, while other symptoms such as feelings of anxiety were more common among the childless. These diverging results for different symptoms help explain the modest overall findings regarding symptoms. Lastly, these results underscore the need to separate symptoms from diagnosed disorders and highlight the importance of examining specific conditions separately. Using a broad mental health measure can obscure real variability.

Our study has several strengths. We have complete coverage of primary health care data and accurate parenthood data for an entire population, meaning no participation bias. Detailed health data also meant we could explore specific diagnoses and symptoms, and identify the diseases or symptoms most strongly associated with parenthood. The fact that our dataset covers the entire population also meant we could look at the relationship between parenthood and mental health at all ages. Finally, due to the richness of the register data, we could use both sibling- and twin-matched designs to limit the confounding influence of genetics and shared family environments.

However, our study also has some limitations. The primary care database does not include services from nursing homes and institutions, likely underestimating disease prevalence in older groups. Additionally, not all primary-care-recorded diagnoses will be confirmed by formal psychiatric evaluations. This is especially important to keep in mind when interpreting our findings for specific diagnoses. Practitioner evaluations might also vary by socioeconomic status or gender, potentially impacting results in ways beyond our control. We also had no way to observe an individual’s fertility intentions, and consequently, we could not separate involuntary and voluntary childlessness. Finally, as our aim was to document mental health differences between groups in society, our conclusions do not depend on assumptions of causality. Nevertheless, the causal effects of parenthood – or of remaining childless – should be addressed in future studies.

Overall, our study found strong links between parenthood and mental health across the life course in the Norwegian context. Parents had markedly better mental health compared to childless individuals, even after adjusting for confounders and when comparing siblings and monozygotic twins. The mental health disparity between fathers and childless men was more pronounced than among women. Policymakers, clinicians, and researchers should consider how childlessness is associated with an increased risk of mental health issues throughout life.

## Supplementary Information

Below is the link to the electronic supplementary material.

Supplementary Material 1

## Data Availability

The data used in this study include primary health care records, demographic information, and educational outcomes for entire cohorts of the Norwegian population. Therefore, the data used in this study is only available by application to the Regional Committees for Medical and Health Research Ethics and the data owners (the Norwegian Directorate of Health and Statistics Norway). The authors cannot share these data with other researchers due to its sensitive nature and potential for identification.

## Footnotes

1 In Norway, very few men and women will have their first child after age 45 [38].

2 The symptom codes cover in total 26 different symptoms, while the diagnosis codes cover 17 conditions. This is because not all codes within the given range exist, e.g. P87-P97 are not in use.
