# Supplementary material for: Parenthood and mental health throughout the life course: A total population study
Source: Soc Psychiatry Psychiatr Epidemiol. 2026 Apr 29;61(9):1867–80. doi: 10.1007/s00127-026-03091-7 (PMC13578102; doi:10.1007/s00127-026-03091-7)
Supplement: Supplementary file 1 [file 127_2026_3091_MOESM1_ESM.pdf]

Appendix A Supplements

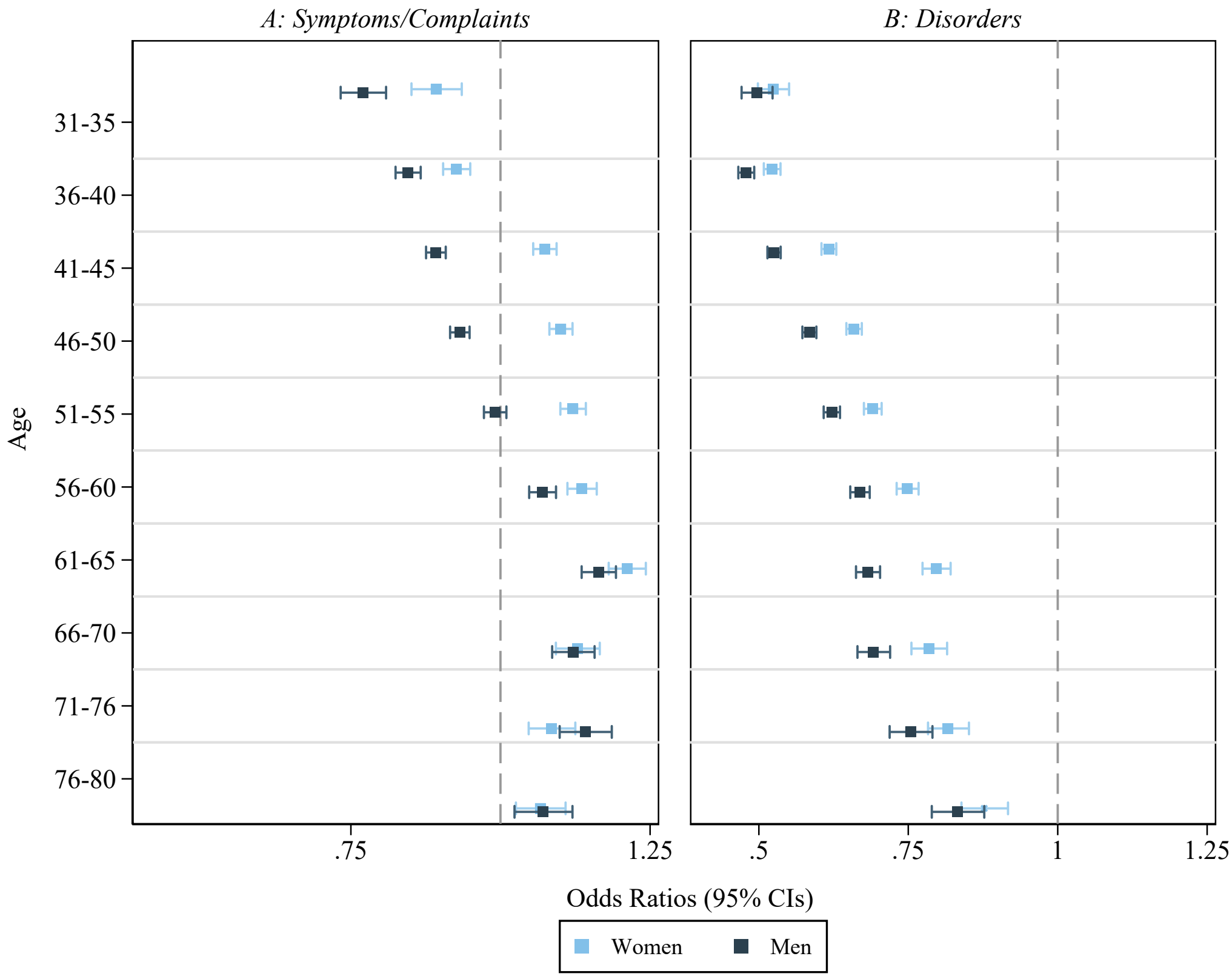

Fig. A1 Estimated odds ratios of psychological symptoms/complaints (A) and mental disorders (B) by parenthood (=1) at age 45, controlling for educational attainment, partnership, age at first birth, and year.

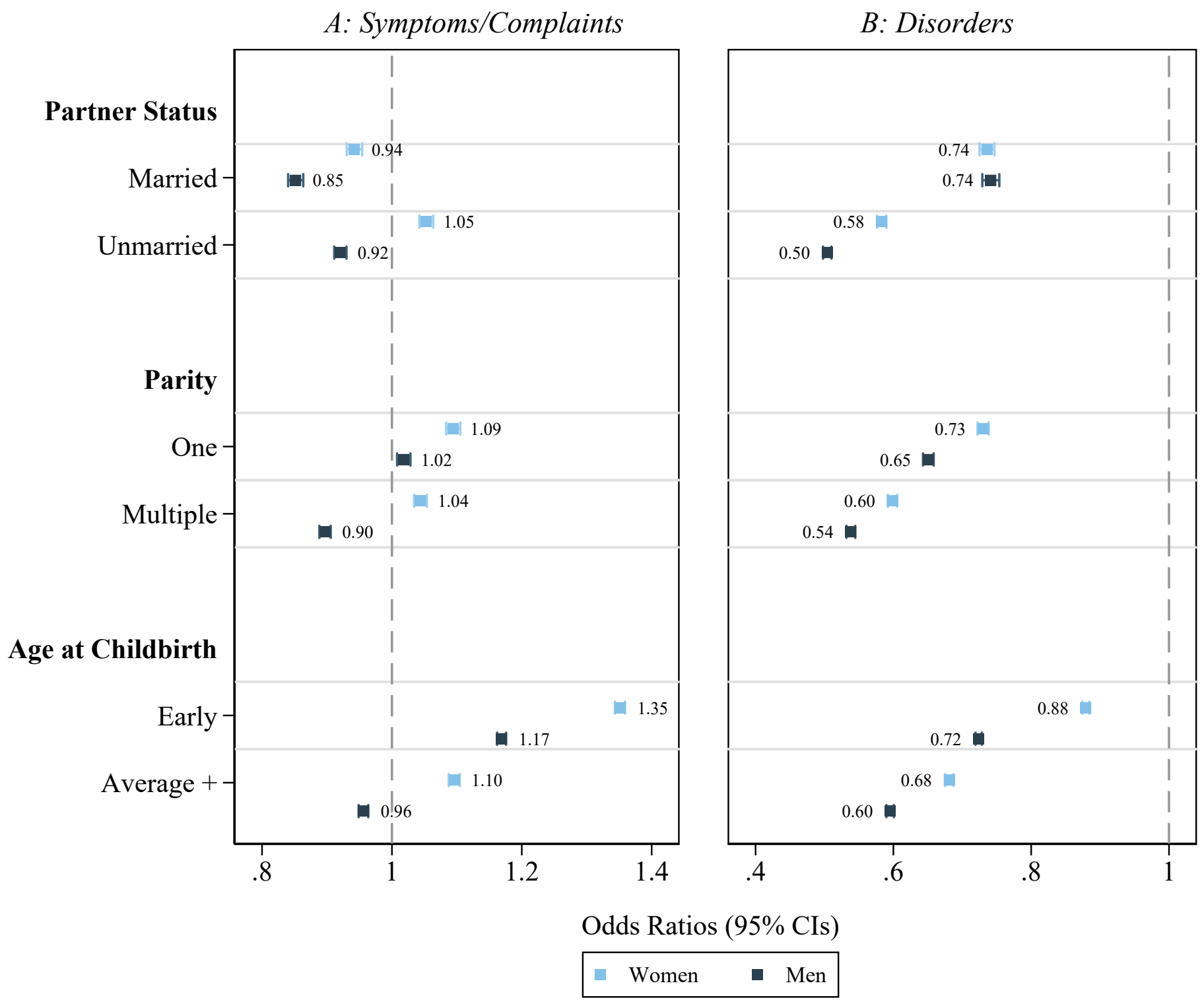

Fig. A2 Estimated odds ratios of psychological symptoms/complaints (A) and mental disorders (B) by parenthood (=1) at age 45, controlling for educational attainment, age, and year. Models also control for the other markers of socioeconomic background not used to limit the sample (i.e. partner status, parity, and age at childbirth).

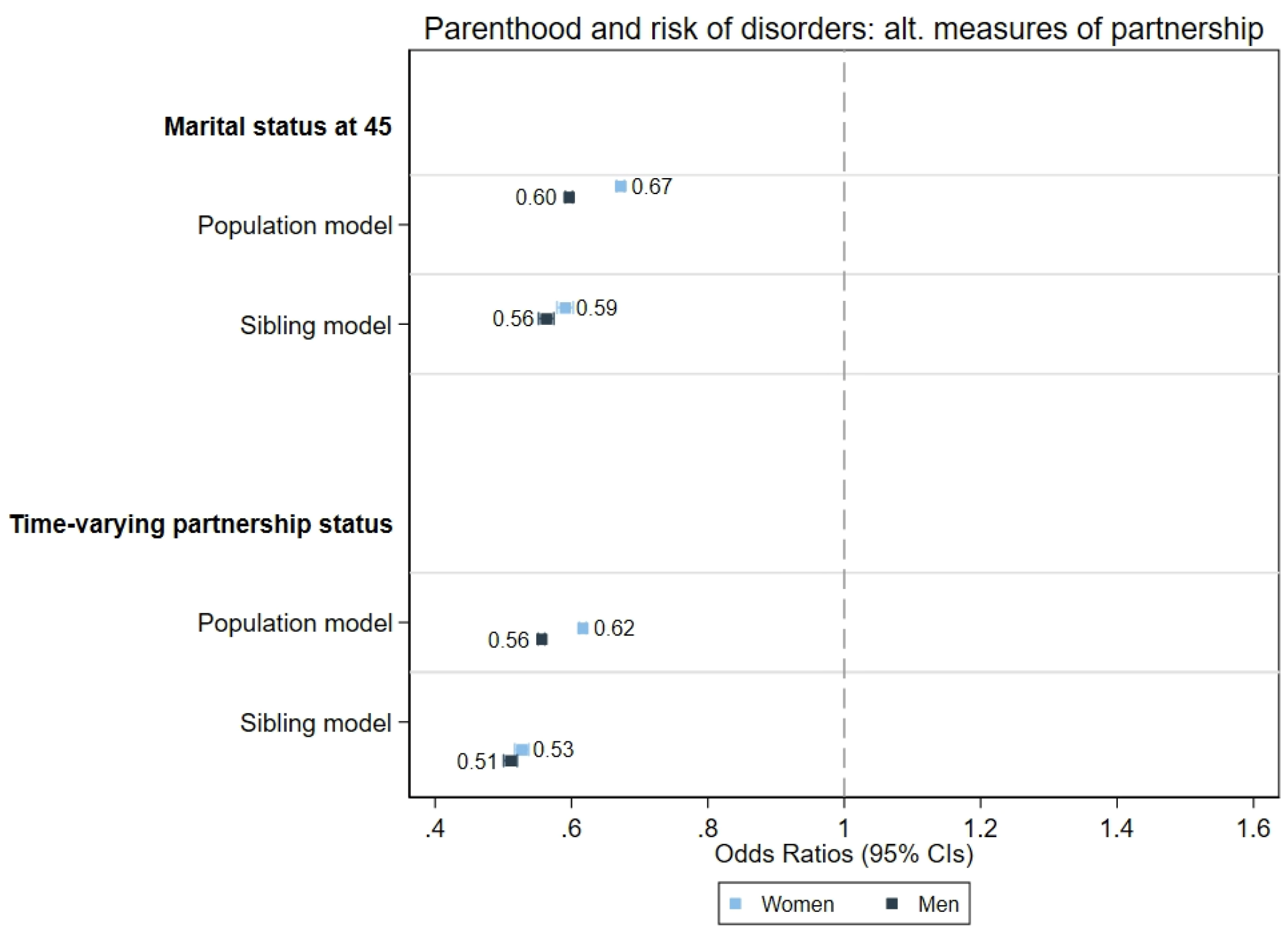

Fig. A3 Summary of the different association models estimated between parenthood (parent=1) and mental disorder risk. Models are specified either with controls for marital status measured at age 45, or measured as a time-varying categorical variable covering all types of registered relationship status (e.g. married, divorced, widowed, separated etc.).

Table A1 Summary Statistics

	2006	2007	2008	2009	2010	2011	2012	2013	2014	2015	2016	2017	2018	2019
**Full sample**														
Age	51.88	52.48	53.10	53.72	54.36	54.99	55.64	56.28	56.96	57.63	58.31	58.98	59.65	60.31
Female	0.50	0.50	0.50	0.50	0.50	0.50	0.50	0.50	0.50	0.50	0.50	0.50	0.50	0.50
Childless at 45	0.13	0.13	0.13	0.13	0.13	0.13	0.13	0.13	0.13	0.13	0.13	0.13	0.13	0.13
Married at 45	0.65	0.65	0.65	0.65	0.65	0.65	0.65	0.64	0.64	0.64	0.64	0.63	0.63	0.63
Age at First Birth	26.46	26.45	26.45	26.45	26.46	26.46	26.48	26.49	26.51	26.53	26.55	26.57	26.60	26.63
Early Age at First Birth	0.42	0.42	0.43	0.43	0.43	0.43	0.42	0.42	0.42	0.42	0.42	0.42	0.42	0.41
Med. Age at First Birth	0.36	0.36	0.36	0.36	0.36	0.36	0.36	0.36	0.36	0.36	0.36	0.36	0.36	0.36
Adv. Age at First Birth	0.09	0.09	0.09	0.09	0.09	0.09	0.09	0.09	0.09	0.09	0.09	0.09	0.09	0.09
Lower Secondary (Compulsory)	0.47	0.46	0.45	0.45	0.44	0.43	0.43	0.42	0.41	0.41	0.40	0.39	0.39	0.38
Upper Secondary	0.25	0.26	0.26	0.26	0.27	0.27	0.27	0.28	0.28	0.28	0.28	0.29	0.29	0.29
Bachelor's Degree	0.21	0.21	0.22	0.22	0.22	0.22	0.23	0.23	0.23	0.23	0.24	0.24	0.24	0.24
Master's Degree	0.07	0.07	0.07	0.07	0.07	0.07	0.07	0.08	0.08	0.08	0.08	0.08	0.08	0.09
Mental Disorders	7.86	7.86	8.18	8.22	8.24	8.17	8.07	8.18	8.1	8	7.9	7.62	7.48	7.43
Psychological Symptoms	8.78	9.14	9.94	10.25	10.55	10.9	11.14	11.64	12.24	12.8	13.12	12.79	12.83	13.08
Observations	2,394,217	2,352,756	2,312,970	2,273,388	2,234,767	2,195,788	2,156,854	2,117,671	2,080,590	2,043,811	2,006,656	1,968,715	1,929,826	1,889,729
**Aged 45**														
Childless (Men)	18.11	18.6	18.8	18.61	19.3	18.93	18.91	19.2	18.81	19.47	19.51	19.65	20	20.28
Childless (Women)	10.94	10.97	11.26	11.12	10.97	10.69	10.93	10.55	11.05	11.06	11.21	11.22	11.19	11.76
Age at First Birth	27.13	27.19	27.32	27.46	27.54	27.67	27.76	27.86	27.98	28.12	28.31	28.51	28.63	28.70
Mental Disorders	8.62	8.53	8.97	8.90	9.06	9.35	9.00	9.20	9.23	9.04	9.24	9.08	9.22	9.66
Psychological Symptoms	9.33	9.55	10.50	11.05	11.07	11.97	11.67	12.18	12.93	13.43	14.00	13.76	13.92	14.63
Observations	57,092	56,916	57,898	59,995	60,466	61,400	61,238	62,396	62,870	60,042	60,939	59,905	57,194	55,694

Summary stats are for the entire sample in that year. For example, age at first birth in 2006 is not calculated only based on those that gave birth in 2006, but for everyone in the sample in 2006.

Table A2 Mental Disorders (ORs)

Variables	(1) W	(2) W	(3) W - Siblings	(4) W - Twins	(5) M	(6) M	(7) M - Siblings	(8) M - Twins
Parent by 45	0.620***	0.672***	0.591***	0.620***	0.445***	0.596***	0.563***	0.715**
	(0.00161)	(0.00293)	-0.00606	(0.0791)	(0.0011)	(0.00271)	(0.00569)	(0.114)
Age		0.988***	0.980***			0.987***	0.978***	
		$(8.94e-05)$	(0.000474)			(0.00011)	(0.000548)	
Married at 45		0.554***	0.527***	0.712***		0.546***	0.514***	0.698***
		(0.00108)	(0.00246)	(0.0440)		(0.0014)	(0.00298)	(0.0653)
Early Age at First Birth		1.289***	1.191***	1.066		1.168***	1.066***	1.055
		(0.00499)	(0.0106)	(0.125)		(0.00512)	(0.0101)	(0.150)
Med. Age at First Birth		1.037***	0.996	1.041		1.017***	0.950***	0.928
		(0.00407)	(0.00874)	(0.112)		(0.00449	(0.0089)	(0.134)
Lower Secondary		2.087***	2.144***	1.713***		2.196***	2.448***	1.304
		(0.0106)	(0.0251)	(0.299)		(0.011)	(0.03)	(0.285)
Upper Secondary		1.434***	1.566***	1.325*		1.336***	1.574***	1.294
		(0.00744)	(0.0183)	(0.225)		(0.00686)	(0.019)	(0.272)
Bachelor's Degree		1.380***	1.433***	1.279*		1.372***	1.491***	1.581**
		(0.00706)	(0.0156)	(0.189)		(0.00746)	(0.0179)	(0.286)
Year		1.002***	1.010***	0.988***		1.010***	1.024***	1.015***
		(0.000237)	(0.000577)	(0.0791)		(0.000294)	(0.00068)	(0.114)
Number of groups	-	-	252,253	1,015	-	-	199,681	582
Observations	14, 449, 405	14, 449, 405	5, 100, 848	25,330	14, 373, 917	14, 373, 917	3,970,448	13,460

Robust standard errors in parentheses
*** p<0.01, ** p<0.05, * p<0.1
Reference groups: older age at first birth, master's degree

Table A3 Psychological Symptoms/Complaints (ORs)

Variables	(1)<br>W	(2)<br>W	(3)<br>W - Siblings	(4)<br>W - Twins	(5)<br>M	(6)<br>M	(7)<br>M - Siblings	(8)<br>M - Twins
Parent by 45	1.031***	1.093***	1.066***	1.194*	0.796***	0.978***	0.983**	0.967
	(0.00268)	(0.00421)	(0.00892)	(0.127)	(0.0019)	(0.0038)	(0.00797)	(0.119)
Age		0.994***	0.990***			0.994***	0.988***	
		(0.0000776)	(0.000387)			(0.0000932)	(0.000435)	
Married at 45		0.673***	0.688***	0.730***		0.628***	0.645***	0.600***
		(0.00113)	(0.00259)	(0.0367)		(0.00134)	(0.00291)	(0.0433)
Early Age at First Birth		1.227***	1.161***	0.953		1.170***	1.074***	1.081
		(0.00389)	(0.00814)	(0.0905)		(0.0041)	(0.00788)	(0.118)
Med. Age at First Birth		1.010***	1.002	0.774***		1.001	0.963***	1.397***
		(0.00321)	(0.00684)	(0.0669)		(0.00352)	(0.00693)	(0.149)
Lower Secondary		1.339***	1.193***	0.646***		1.790***	1.692***	1.083
		(0.0053)	(0.0106)	(0.0928)		(0.00715)	(0.016)	(0.181)
Upper Secondary		1.166***	1.134***	0.670***		1.331***	1.359***	0.984
		(0.00471)	(0.00997)	(0.0928)		(0.0054)	(0.0125)	(0.160)
Bachelor's Degree		1.242***	1.195***	0.983		1.321***	1.324***	1.050
		(0.0049)	(0.00967)	(0.115)		(0.00569)	(0.012)	(0.150)
Year		1.040***	1.053***	1.054***		1.043***	1.063***	1.050***
		(0.000211)	(0.000484)	(0.00378)		(0.000255)	(0.000554)	(0.00502)
Number of groups	-	-	394,943	1,589	-	-	337,360	1,121
Observations	14, 449, 405	14, 449, 405	7, 955, 264	39,375	14, 373, 917	14, 373, 917	6, 942, 125	26,743

Robust standard errors in parentheses
*** p<0.01, ** p<0.05, * p<0.1
Reference groups: older age at first birth, master's degree
